# Major Virion Tegument Protein VP22 Targets Nuclear Matrix and Chromatin upon Entry into Cells during Productive Herpes Simplex Virus 1 Infection

**DOI:** 10.3390/microorganisms12030521

**Published:** 2024-03-05

**Authors:** Isabella Chi, John A. Blaho

**Affiliations:** 1 Department of Microbiology, Mount Sinai School of Medicine, New York, NY 10029, USA; 2 Medical Diagnostic Laboratories LLC, Hamilton, NJ 08690, USA; 3 Innovation and Applied Research Division, City University of New York, New York, NY 10031, USA

**Keywords:** HSV-1, virion VP22, phosphorylation, nuclear matrix, chromatin

## Abstract

HSV-1 major tegument protein VP22 is present in multiple subcellular locations in the late stages of productive viral infection. We initially performed a detailed time course experiment and observed that VP22 was detected in nuclear and nuclear matrix fractions as early as 4 hpi. The goal was to determine the fate of virion-derived incoming VP22, and we report the following: (i) VP22 was detected in nuclear matrix fractions 1 hpi. (ii) In the presence of cycloheximide (CHX), VP22 was present in the nuclear matrix 1–6 hpi, demonstrating the stability of the protein. (iii) The nuclear matrix targeting of VP22 occurred in infected Vero, HEp-2, and human mammary epithelial (HME) cells and following synchronized infection. Based on these results, we conclude that (iv) VP22 targets the nuclear matrix and chromatin upon entry into cells during productive HSV-1 infection.

## 1. Introduction

Herpes simplex virus (HSV) infections occur worldwide, and the virus is only transmitted between humans (reviewed in [1,2]). Once HSV has infected an individual, it remains in their body for life, hidden in a dormant state inside the trigeminal ganglion. Most of what is known about the replicative cycle of HSV comes from work in tissue culture systems using herpes simplex virus 1 (HSV-1). The expression of HSV-1 genes is coordinately regulated and ordered in a sequential cascade [3,4]. The first class of genes which are expressed after infection are the immediate early (IE) genes. These genes do not require de novo viral protein synthesis for their expression [5,6] and are induced by a structural component of the virion, VP16 [5,7,8]. Expression of the later classes of genes, early and late, requires functional IE proteins [9,10]. The exact molecular mechanism of HSV-1 tegument and envelope assembly is poorly understood. Most of the major tegument proteins, especially VP22, exhibit nuclear and cytoplasmic distributions late in infection [11,12,13,14,15,16,17,18,19], and there is general agreement that primary envelopment occurs as the capsid exits the nucleus [20,21,22,23,24,25,26,27,28].

VP22 is one of the most abundant tegument proteins in HSV-1 virus particles, contributing almost ten percent of the mass of the virion [29]. However, the role of VP22 during HSV-1 infection remains unclear (recently reviewed in [30]). Full-length VP22 is required for efficient viral cell-to-cell spread during infection in animals and cultured cells [31,32]. It must be recognized that such a growth defect cannot discriminate between early or late functions of the VP22. In general, it appears that most data on VP22 activity may be consistent with a function of the protein during virus assembly/maturation and egress [22,31,33,34,35,36,37,38,39,40].

Interest in VP22 has recently been reinvigorated following observations that it associates with mRNA and may play a role in protein synthesis late in infection [40,41,42]. VP22 has also been implicated in blocking the immune response during infection [43,44,45,46], though these mechanisms of action remain unclear. VP22 of HSV-1 possesses two nuclear localization signals (NLSs) [47], and the protein accumulates to high levels in the nuclei of either infected cells [17,18,48,49] or VP22-expressing stable cell lines [32]. VP22 also associates with dispersed nucleoli and marginalized chromatin during productive infection [50]. These data, along with the report that VP22 can bind RNA and possibly transport RNA from one cell to another [51], suggest the possibility of an earlier function of VP22.

To gain greater insight into the behavior and properties of VP22 during infection, we performed a detailed infection time course analysis in which biochemical subcellular fractionations were performed and characterized using specific protein controls. VP22 was detected in nuclear and nuclear matrix fractions 4 hpi. In the presence of cycloheximide (CHX), VP22 was present in the nuclear matrix 1–6 hpi, demonstrating the stability of the protein. Taking these results together, it appears that VP22 targets nuclear matrix and chromatin upon entry into cells during productive HSV-1 infection. These findings represent a significant new feature of VP22 biology which will likely have an important impact on HSV-1 replication research.

## 2. Materials and Methods

### 2.1. Cells and Viruses

African green monkey kidney Vero cells and human epithelial HEp-2 cells were obtained from the American Type Culture Collection (Manassas, VA, USA) and maintained in Dulbecco’s modified Eagle medium (DMEM) supplemented with 5% fetal bovine serum. Human mammary epithelial (HME) cells were obtained from Clonetics (San Diego, CA, USA) and maintained as previously described [52]. A very-low-passage original isolate of HSV-1(KOS) virus is the prototype wild-type strain used in our studies and was generously provided by Priscilla Schaffer (University of Arizona) and plaque-purified by us. To obtain virus stocks, subconfluent monolayer Vero cells (4 × 10^6^ cells) were inoculated at a multiplicity of infection (MOI) of 0.01 for 2 h at 37 °C in DMEM supplemented with 2% newborn calf serum. The inoculum was then removed; fresh DMEM supplemented with 5% newborn calf serum was added, and cells were incubated at 37 °C in 5% CO_2_ for 2–3 days. Virus stocks were prepared once the infection reached a cytopathic effect of 100%; the virus titers on Vero cells were determined, and aliquots were stored at −80 °C. All the MOIs were derived from the number of PFUs on Vero cells.

### 2.2. Synchronized Infection

Synchronized infection is defined as uniform staining in all cells in a microscopic field at a given time post infection, as determined by indirect immunofluorescence with specific antibodies for unique HSV-1 polypeptides [18]. Vero cells were grown the day before infection in 25 cm^2^ dishes. Cells were incubated on ice at 4 °C for 20 min prior to the addition of virus. After the virus was allowed to adsorb for 1 h, the cells were removed from the ice, and medium preheated to 37 °C was added immediately; then, cells were returned to a 37 °C incubator. Induction of synchronous infection by adsorption of the inoculum at 4 °C is routine and has little or no effect on cells in culture [18].

### 2.3. Chemical Treatment during Infection

To inhibit de novo protein synthesis, cycloheximide (CHX; Sigma, St. Louis, MO, USA) at a final concentration of 10 mg/mL was added to the medium 30 min prior to infection. This concentration was previously shown to be sufficient to completely block viral protein synthesis in HSV-1-infected Vero and HEp-2 cells [53]. CHX was maintained in the medium at this concentration until the infection was terminated.

### 2.4. Infected Cell Extracts

Extracts were prepared using a modification of previous procedures [18,54,55,56]. Approximately 2 × 10^6^ infected cells were harvested directly into the medium by gentle scraping; briefly pelleted by low-speed centrifugation; and washed once in 140 mM NaCl, 3 mM KCl, 10 mM Na_2_HPO_4,_ and 1.5 mM KH_2_PO_4_ at pH 7.5 (PBS). The following protease inhibitors were added to PBS and all other solutions used therein: 10 mM L-1-chlor-3-(4-tosylamido)-7-amino-2-heptanon-hydrochloride (TLCK), 10 mM L-1-chlor-3 (4-tosylamido)-4-phenyl-2-butanone (TPCK), and 100 mM phenylmethylsulfonyl fluoride (PMSF). Whole-cell extracts were prepared by adding 300 mL of 10 mM Tris-HCl (pH 8.0), 1.5 mM MgCl_2_, 10 mM KCl, and 0.5 mM DTT (Dignam buffer A), followed directly by sonication using a Branson sonifier. For subcellular fractionations, 300 mL of Digman buffer A was added to cells, and they were incubated on ice for 5 min to swell. The cells were lysed through several (6) passages with a 25 gauge needle (Becton Dickerson, Franklin Lakes, NJ, USA) followed by centrifugation (6000× *g*) at 4 °C. The supernatant was removed and saved as the cytoplasmic fraction. The pellet (nuclei) was resuspended in 250 mL of 20 mM Tris-HCl (pH 8.0), 420 mM NaCl, 1.5 mM MgCl_2_, 0.2 mM EDTA, and 0.5 mM DTT (Dignam buffer D) and rotated at 4 °C for 30 min prior to centrifugation (6000× *g*) at 4 °C for 5 min. The supernatant was removed and saved as the nuclear extract. The pellet (nuclear matrix) was resuspended in 250 mL of Dignam buffer D, followed directly by sonication using a Branson sonifier. Protein concentrations of infected cell extracts were determined using a modified Bradford assay (Bio-Rad, Hercules, CA, USA) according to the manufacturer’s specifications.

### 2.5. Virion Preparations

Preparations of purified extracellular virions from medium were performed as a modification of methods described previously [32,55]. Approximately 4 × 10^8^ of HSV-1(KOS)-infected Vero cells (MOI = 0.1) were harvested 48 hpi directly into medium by gentle scraping and pelleted by low-speed centrifugation (2000× *g*). The medium was removed and cleared again by high-speed centrifugation (30,000× *g*) using an SW41 swinging bucket rotor (Beckman, Brea, CA, USA). The pellet was resuspended in 25 mM Tris-HCl (pH 7.6), 25 mM EDTA, and 100 mM NaCl (TNE buffer). Virions were loaded onto the top of a sucrose step (10:30:60) gradient, which was previously allowed to equilibrate for 12 h at 4 °C, and ultracentrifuged (60,000× *g*) for 1 h using an SW41 rotor. The virion band which formed approximately between the 30% and 60% interfaces was collected and diluted 10-fold in TNE buffer. Virions were pelleted by ultracentrifugation (60,000× *g*) for 1 h using an SW41 rotor. Pelleted virions were resuspended in TNE buffer, aliquotted, and stored at −80 °C. Virion preparations were tested for the absence of contaminating protein by immunoblotting for cellular tubulin and viral ICP27; no anti-tubulin or anti-ICP27 immune reactivity was detected in any virion preparation.

### 2.6. Phosphatase Treatments

Approximately 50 mg of HSV-1(KOS)-infected (MOI = 10) Vero whole-cell extract protein was reacted (200 mL) with calf intestinal phosphatase (CIP) or protein lambda phosphatase (PLP) (New England Biolabs, Ipswitch, MA, USA) in 50 mM Tris-HCl (pH 8.0), 100 mM NaCl, 10 mM MgCl_2_, and 1 mM dithiothreitol for 30 min at 37 °C. Reactions were terminated by the addition of SDS at 2% and boiling for 5 min. In controls, phosphatase inhibitor cocktail (Sigma) with the addition of EDTA to 50 mM was also added, or no phosphatase was added.

### 2.7. Denaturing Gel Electrophoresis and Immunoblotting

Equal amounts (approximately 150 μg) of infected cell protein were separated in long (25 cm) 15% SDS-polyacrylamide gels cross-linked with N, N’-diallyltartardiamide (DATD), electrically transferred to nitrocellulose, and probed with relevant primary antibodies. Alkaline phosphatase-conjugated goat anti-rabbit and anti-mouse secondary antibodies (Southern Biotech, Birmingham, AL, USA) were used at 1:1000 in PBS containing 5% milk. Pre-stained molecular weight markers (Gibco-BRL, Waltham, MA, USA) were included in all gels.

### 2.8. Immunological Reagents

Rabbit polyclonal antibody RGST49 specific for VP22 [18] was used at a dilution of 1:1000 for immunoblotting. Monoclonal antibody 22-3 specific for VP22 [57,58] was used at a dilution of 1:500 for indirect immunofluorescence. Polyclonal antibody specific for lamin B (SC-6216; Santa Cruz Biotechnology, Inc., Dallas, TX, USA) was used at a dilution of 1:1000. Monoclonal antibody specific for tubulin (DM-A1; Sigma) was used at a dilution of 1:1000. Fluorescein isothiocyanate (FITC)-conjugated anti-mouse immunoglobulin G (IgG heavy plus light chains) and Texas Red-conjugated anti-rabbit IgG were purchased from Molecular Probes, Inc. (Eugene, OR, USA), and used at a dilution of 1:1000. All antibody dilutions were made in 1% bovine serum albumin (BSA; Sigma) in PBS.

## 3. Results

### 3.1. VP22 Detection in Nuclei and Nuclear Matrix 4 hpi

Previous studies by us described the accumulation of VP22 in nuclei in late infection stages [18]. We revisited the behavior of VP22 during the course of an HSV-1 infection biochemically by implementing high-salt extraction of nuclei and utilizing specific cellular protein markers as quality controls. Vero cells were non-synchronously mock-infected and HSV-1(KOS)-infected; fractionated into cytoplasmic, nuclear, and nuclear matrix extracts between 4 and 24 hpi; separated on denaturing gels; transferred to nitrocellulose; and immunoblotted for VP22. Whole-cell extracts were also loaded as starting material controls. As additional controls, lamin B and tubulin antibodies were used to assess the integrity of the cytoplasmic and nuclear matrix fractions, respectively.

**Figure 1:**
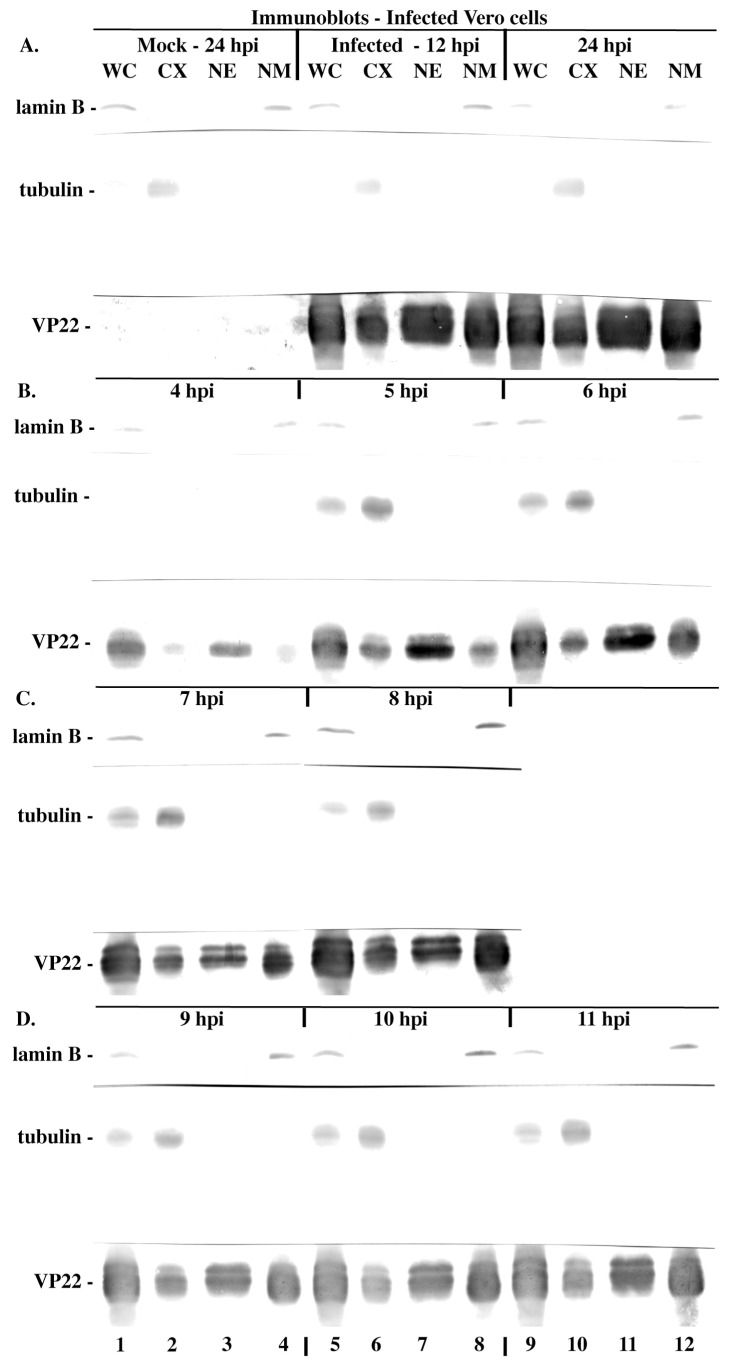
Immune reactivity of infected cell proteins during HSV-1(KOS) infection (**A**–**D**). Vero cells were mock- and HSV-1(KOS)-infected (MOI = 10), and whole-cell (WC), cytoplasmic (CX), nuclear (NX), and nuclear matrix (NM) extracts were prepared each h at the times indicated. Extracted proteins were separated in denaturing gels, transferred to nitrocellulose, and immunoblotted for VP22 as described in Materials and Methods. Tubulin and lamin B were markers for cytoplasm and nuclear matrix, respectively.

VP22 was distributed between the cytoplasmic, nuclear, and nuclear matrix fractions and this overall pattern did not differ between 12 and 24 hpi (in Figure 1A, compare lanes 5–8 with 9–12). The inspection of the fractions between 7 and 11 hpi indicated that while the overall amounts of VP22 were lower at this time than between 12 and 24 hpi, the distributions of the three fractions were again similar (Figure 1C,D, lanes 1–12). At least three different electrophoretic forms of VP22 were detected in infected cells but not in mock-infected cells, consistently with previous studies [18]. In contrast, 4–6 hpi, VP22 predominated in the nuclear and nuclear matrix fractions, compared with the cytoplasmic fraction (Figure 1B, lanes 1–12). As expected, the cytoplasmic fraction contained control tubulin staining, while the nuclear matrix fraction contained control lamin B, confirming the integrity of our fractions. As there was no obvious VP22 signal 4 h post infection, but a very obvious signal was present 5 hpi, this indicates that VP22 made de novo is virtually the only detected species in these early stages under these experimental conditions. Based on these results, we conclude that in early infection stages (<6 hpi), the VP22 inside infected cells resides mainly in the nucleus, with a significant proportion of VP22 remaining in the nuclear matrix after high-salt extraction.

### 3.2. Incoming VP22 Targets the Nuclear Matrix

The previous results indicate an early nuclear accumulation of VP22 during HSV-1 infection. We next set out to determine whether this VP22 was newly synthesized or derived from incoming virions. Vero cells were mock- and HSV-1(KOS)-infected in the presence and absence of CHX during synchronized infection; fractionated into cytoplasmic, nuclear, and nuclear matrix extracts between 1 and 6 hpi; separated on denaturing gels; transferred to nitrocellulose; and immunoblotted for VP22, and control tubulin and lamin B.

**Figure 2:**
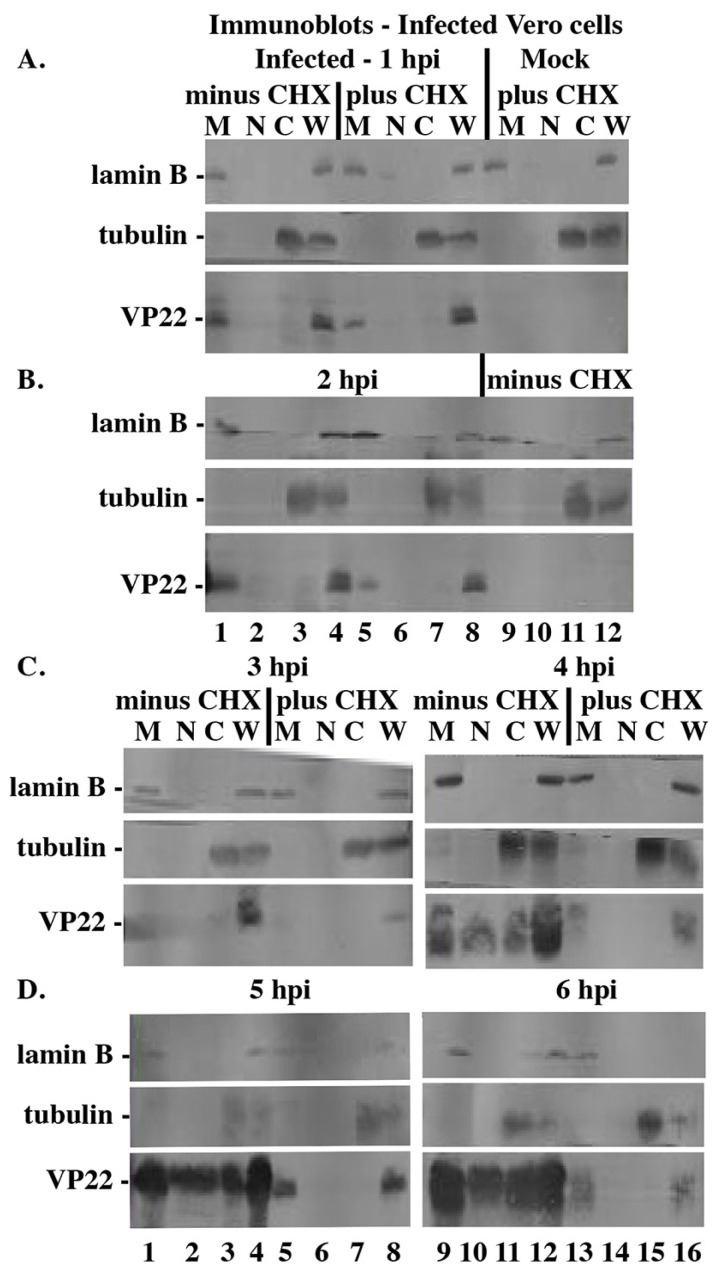
Immune reactivity of infected cell proteins during HSV-1(KOS) infection (Panels (**A**–**D**)). Vero cells were synchronously mock- and HSV-1(KOS)-infected (MOI = 10) in the presence (plus) or absence (minus) of CHX, and whole-cell (W), cytoplasmic (C), nuclear (N), and nuclear matrix (M) extracts were prepared each h at the times indicated. Extracted proteins were separated in denaturing gels, transferred to nitrocellulose, and immunoblotted for VP22 as described in Materials and Methods. Tubulin and lamin B were markers for cytoplasm and nuclear matrix, respectively. Mock extracts were prepared 4 hpi.

At 1 and 2 hpi, VP22 was detected in the nuclear matrix fraction both in the absence and presence of CHX (in Figure 2A, lanes 1 and 5; in Figure 2B, lanes 1 and 4). Thus, the observed behavior of VP22 in these extremely early stages was not simply a consequence of CHX treatment. In the presence of CHX, VP22 was only detected in the nuclear matrix fraction 4, 5, and 6 hpi (in Figure 2C, lane 13; in Figure 2D, lanes 5 and 13, respectively). Based on these results, we conclude that incoming VP22 directly targets the nuclear matrix. In addition, this incoming VP22 remains stable and does not readily degrade, inasmuch as it continues to be detected for as long as 6 hpi under CHX treatment.

As expected (Figure 1B), VP22 was detected in the cytoplasmic and nuclear fractions 4–6 hpi in the absence of CHX (in Figure 2C, lanes 10 and 11; in Figure 2D, lanes 2, 3, 10, and 11). These findings suggest that de novo synthesized VP22 is capable of migrating between the cytoplasm and nucleus, while incoming VP22 remains associated with the nuclear matrix.

### 3.3. VP22 Targeting of Nuclear Matrix in Human Mammary Epithelial (HME) and HEp-2 Cells

The studies above (Figure 1 and Figure 2) and our previous studies [18] were performed exclusively in immortalized African green monkey kidney cells (Vero). It was, therefore, important to determine whether the virion VP22 targeting of the nuclear matrix was cell-type-specific. Two sets of experiments were performed. In the first series, immortalized human mammary epithelial (HME) cells were infected, and subcellular fractions were prepared 24 hpi. In the second series, human epithelial HEp-2 cells were infected in the presence or absence of CHX, and subcellular fractions were prepared 3 hpi. Infected cell proteins were separated in denaturing gels, transferred to nitrocellulose, and immunoblotted for VP22. In the second set of experiments, the Ponceau S (Sigma) staining of nitrocellulose prior to immunoblotting confirmed the integrity of the fractions.

**Figure 3:**
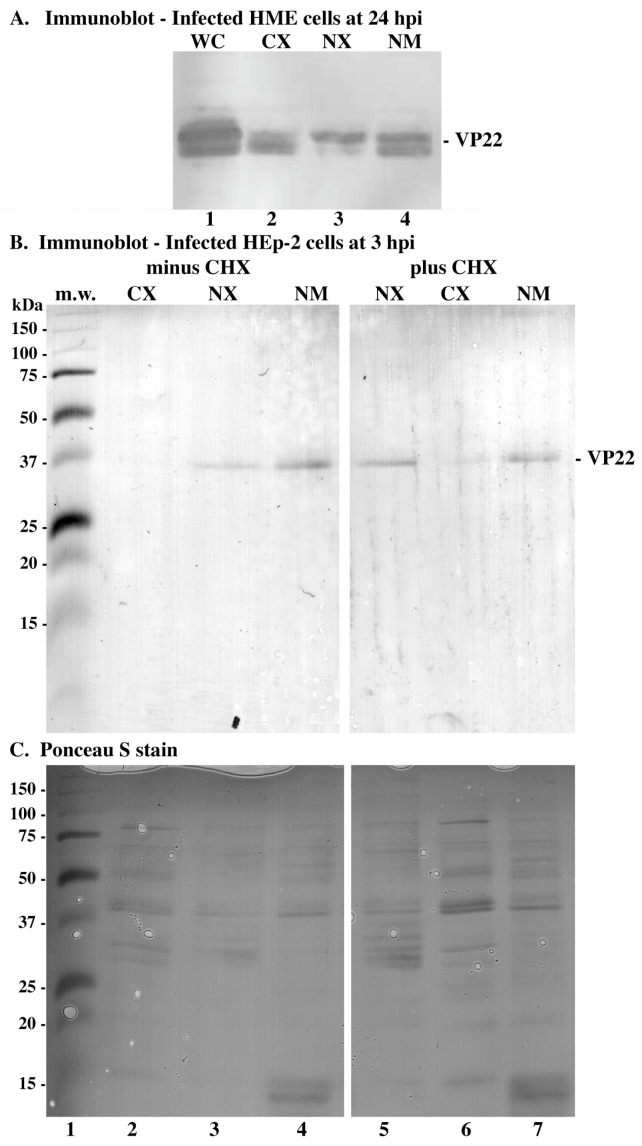
Immune reactivity (**A**,**B**) and dye staining (**C**) of infected cell proteins during HSV-1(KOS) infection. HME cells (**A**) were mock- and HSV-1(KOS)-infected (MOI = 10) only, and HEp-2 (**B**,**C**) cells were mock- and HSV-1(KOS)-infected (MOI = 10) in the presence (plus) or absence (minus) of CHX. Whole-cell (WC), cytoplasmic (CX), nuclear (NX), and nuclear matrix (NM) extracts were prepared at the times indicated. Extracted proteins were separated in denaturing gels, transferred to nitrocellulose, and immunoblotted for VP22 as described in Materials and Methods. Ponceau S staining occurred prior to immunoblotting. Migration of mol wt markers is indicated in the left margins.

Consistent with the results above (Figure 1 and Figure 2), at least three isoforms of VP22 were detected in the fractions of infected HME cells 24 hpi (in Figure 3A, lane 1). The observation that, in this very late infection stage, cytoplasmic VP22 was mainly fast migrating while nuclear extract VP22 was slower migrating is consistent with previous observations at 24 hpi in Vero cells [18]. We observed a significant amount of VP22 within the nuclear matrix fraction of infected HME cells (in Figure 3A, compare lanes 4 with 2 and 3). Based on these results, we conclude that similar to Vero cells, in infected human mammary epithelial cells, VP22 associates with the nuclear matrix in late infection stages.

In infected HEp-2 cells, at 3 hpi, VP22 was detected in the nuclear matrix fraction as well as the nuclear fraction both with and without CHX (Figure 3B). Although this differs slightly from the findings in Vero cells (Figure 2), the key observation is that incoming VP22 is in infected HEp-2 nuclei 3 hpi. The detection of VP22 in the nuclear fraction may imply that infected HEp-2 nuclei are more fragile or sensitive to biochemical manipulations than infected Vero nuclei. Consistent with this idea is the fact that there was more nuclear VP22 in the CHX-treated nuclear fraction than in that without CHX (in Figure 3B, compare lanes 5 with 3). Inspection of the Ponceau S staining patterns (Figure 3C) does not indicate any obvious contamination among the fractions, so the nuclear localization of VP22 under these conditions may be specific to this viral protein. Overall, based on these findings, we conclude that the nuclear matrix targeting of incoming VP22 is not cell-type-specific, inasmuch as it occurs in infected Vero, HME, and HEp-2 cells. Thus, this behavior is likely an intrinsic property of virion-derived VP22.

### 3.4. Nuclear Matrix Targeting of Fast Migrating VP22 during Synchronized Vero Cell Infection

Our previous analyses of the subcellular localizations of VP22 were performed using synchronously infected Vero cells [18,47,50,59]. Vero cells were synchronously infected in the presence or absence of CHX, and subcellular fractionations were performed 0.5, 2, and 3 hpi, prior to immunoblotting for VP22, and control tubulin and lamin B.

**Figure 4:**
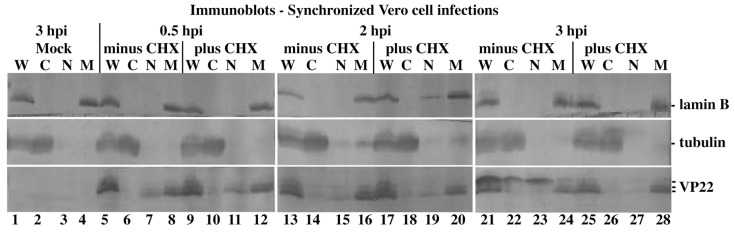
Immune reactivity of infected cell proteins during synchronized HSV-1(KOS) infection. Vero cells were synchronously mock- and HSV-1(KOS)-infected (MOI = 10) in the presence (plus) or absence (minus) of CHX, and whole-cell (W), cytoplasmic (C), nuclear (N), and nuclear matrix (M) extracts were prepared at the times indicated. Extracted proteins were separated in denaturing gels, transferred to nitrocellulose, and immunoblotted for VP22 as described in Materials and Methods. Tubulin and lamin B were markers for cytoplasm and nuclear matrix, respectively. Mock extracts were prepared 3 hpi.

Consistently with the results in Figure 2, VP22 was detected in the nuclear matrix fraction between 0.5 and 3 hpi both without (in Figure 4, lanes 8, 16, and 24) and with CHX (in Figure 4, lanes 12, 20, and 28). VP22 was detected in the nuclear fractions both with and without CHX 30 min post infection (in Figure 4, lanes 7 and 11). This may represent the transient translocation of incoming VP22 from virions on the cell surface to nuclei, and the process appears to be complete by 2 hpi in synchronous infection (in Figure 4, lanes 15 and 19). VP22 detected 3 hpi without CHX in synchronous infection, which was presumably de novo synthesized, was present in cytoplasmic and nuclear extracts as slow-migrating forms (in Figure 4, lanes 22 and 23). Such slow-migrating VP22 forms were previously observed in nuclear fractions 5 hpi under similar conditions, but their origin was unknown [18]. It is conceivable that these forms also represent transient but newly synthesized forms of VP22. Taken together, we conclude that incoming VP22 targets the nuclear matrix during synchronized HSV-1 infection.

### 3.5. Slow-Migrating VP22 Isoform Sensitivity to Phosphatase Treatment

Throughout the previous studies (Figure 1, Figure 2, Figure 3 and Figure 4), we observed at least three different electrophoretic forms of VP22. In previous studies, we showed that all forms of VP22, except the fastest-migrating one, incorporate radiolabeled phosphate [18,32,55]. The goal of this portion of the study was to determine the sensitivity of VP22 to phosphatase treatment. Whole-cell extracts of HSV-1(KOS)-infected Vero cells were prepared 24 hpi and were either left untreated or treated with calf intestinal (CIP) or protein lambda (PLP) phosphatase in the presence or absence of phosphatase inhibitor (Sigma). As controls, extracts were incubated in phosphatase reaction buffer without the enzymes. In addition, virions were prepared from HSV-1(KOS)-infected Vero cells 24 hpi. The extracted proteins were separated in denaturing gels, transferred to nitrocellulose, and immunoblotted for VP22.

**Figure 5:**
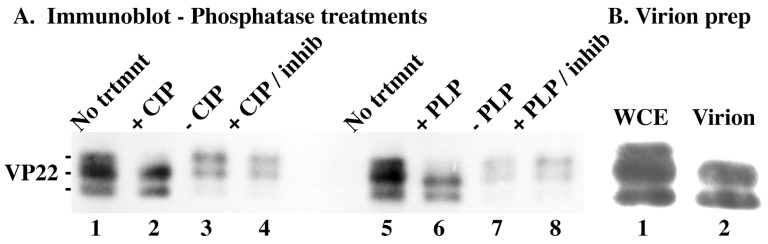
Immune reactivity of infected cell (**A**) and virion (**B**) proteins. (**A**) Vero cells were HSV-1(KOS)-infected for 24 h (MOI = 10), whole-cell extracts were prepared and either left untreated or treated (+) with CIP, PLP, or CIP/PLP in the presence of phosphatase inhibitor (inhib). As controls, extracts were incubated in CIP/PLP reaction buffer without enzymes (−). (**B**) Virions were prepared from HSV-1(KOS)-infected Vero cells 24 hpi as described in Materials and Methods. The extracted proteins were separated in denaturing gels, transferred to nitrocellulose, and immunoblotted for VP22 as described in Materials and Methods.

Treatment with either phosphatase resulted in a reduction in the slowest-migrating forms of VP22 compared with the no-treatment controls (in Figure 5A, compare lanes 2 with 1, and 6 with 5). This reduction was not a result of proteolytic degradation, since it did not occur either in the presence of inhibitors or without phosphatase (in Figure 5A, lanes 4 and 8 and 3 and 7, respectively). The comparison of VP22 derived from untreated whole-cell extract with that from purified virions indicated that phosphatase-treated VP22 (in Figure 5A, lanes 2 and 6) had similar electrophoretic mobility patterns to virion VP22 (in Figure 5B, lane 2). Based on these results, we conclude the following: The slower-migrating forms of VP22 are phosphorylated, inasmuch as they are sensitive to phosphatase treatment. In addition, the virion form of VP22, which has a faster-migrating appearance, is likely due to “underphosphorylation”.

## 4. Discussion

Previous studies investigating the biological function of HSV-1 major tegument protein VP22 mainly focused on activities putatively associated with virion assembly and maturation. In this study, we evaluated the biochemical properties and behavior of VP22 in infected cells immediately upon its entry into infected cells. Our key findings may be summarized as follows.

### 4.1. Incoming Virion-Derived VP22 Targets the Nuclear Matrix

This conclusion is based on our biochemical subcellular fractionation studies. It is important to recognize that in contrast to our earlier fractionation work, which utilized physiological salt conditions along with sonication [18], the current data were derived from Dignam high-salt extractions [54]. Thus, VP22 that remained bound to the matrix could not be removed in 0.4 M NaCl, suggesting tight binding. Earlier studies did indeed detect very low levels of nuclei VP22 5 h post synchronous infection [18]. This earlier-observed VP22 most likely represents de novo synthesized VP22. This conclusion is based on our current data, which show slow electrophoretic VP22 forms in nuclear and cytoplasmic fractions 3 hpi in the absence of CHX (Figure 4). Taking all of these findings together, one immediately realizes that VP22’s behavior in infected cells in early infection stages is much more complicated than originally expected. What seems clear now is that virion-derived VP22 directly enters the nucleus and partitions with the nuclear matrix. Our data suggest that this form of VP22 has high electrophoretic mobility (Figure 4). When newly synthesized VP22 is produced, it can be detected in the cytoplasm and nucleus, consistently with earlier findings [18]. Obvious questions which remain to be elucidated include the role of post-translational modifications in these early subcellular localizations of VP22 and whether the nuclear matrix targeting by VP22 is a regulated process.

### 4.2. Slow-Migrating Isoforms of VP22 Are Sensitive to Phosphatase Treatment

As just noted above, the post-translational modification of VP22 likely plays a significant role in determining/regulating the subcellular localizations of VP22 throughout the course of productive HSV-1 infection. Our data showing that the slow electrophoretic isoforms of VP22 are sensitive to phosphatase treatment are important because the post-treatment forms of VP22 migrate in an essentially identical manner to virion-derived VP22 (Figure 5). It is intriguing that the forms of VP22 that directly target the nuclear matrix also have a similar migration pattern to virion and phosphatase-treated VP22 (compare Figure 4 and Figure 5). It is tempting to speculate that immediately upon infection, “undermodified” virion-derived VP22 enters cells and directly targets the nuclear matrix. Thus, if the post-translational processing of VP22 occurs at this time, it is likely to be transient in nature.

### 4.3. Incoming VP22 Associates with Chromatin

While the biochemical results are compelling, we recognize that they are limited by that fact that they can only track VP22 to the nuclear matrix fraction. Previous studies by several investigators have provided circumstantial evidence for an association of VP22 with chromatin [60,61,62]. Both transfected VP22 and VP22 in stable cell lines were shown to bind the chromatin of dividing cells [32,63]. Under unique infection conditions, in which infected cells might actually proceed through cell cycle, VP22 was reported to be associated with chromatin [48]. O’Hare’s group showed that VP22 binds Template-Activating Factor I (TAF-I) alpha and beta and that this interaction with TAF-I may inhibit the TAF-I–chromatin assembly activity in vitro [64]. Finally, the bovine herpes virus 1 homologue of VP22, bVP22, was shown to associate with mitotic chromosomes, as well as purified histones [65]. Our contribution to this discussion is that we now observe incoming virion-derived VP22 targeting chromatin, perhaps unifying all of these prior observations.

## 5. Conclusions

### 5.1. The Most Important Question Remaining Is what Is the Function of this Early Association of VP22 with Chromatin

At least two potential explanations may exist. The first involves the direct association of VP22 with cellular histones. It is conceivable that VP22 might function to “extract” histones to be used in the chromatinization of the incoming viral genome [61]. The almost immediate detection of VP22 in the nuclear matrix would support this model. Alternatively, VP22 may serve an inhibitory function to squelch the immediate host response to viral infection. This effect would likely occur at the level of suppressing cellular transcription at regions of active chromatin. The development of appropriate biochemical and molecular genetic systems is required to address these and other key questions regarding the function of VP22’s association with chromatin.

### 5.2. Modified Model of VP22 Function during Productive HSV-1 Infection

**Figure 6:**
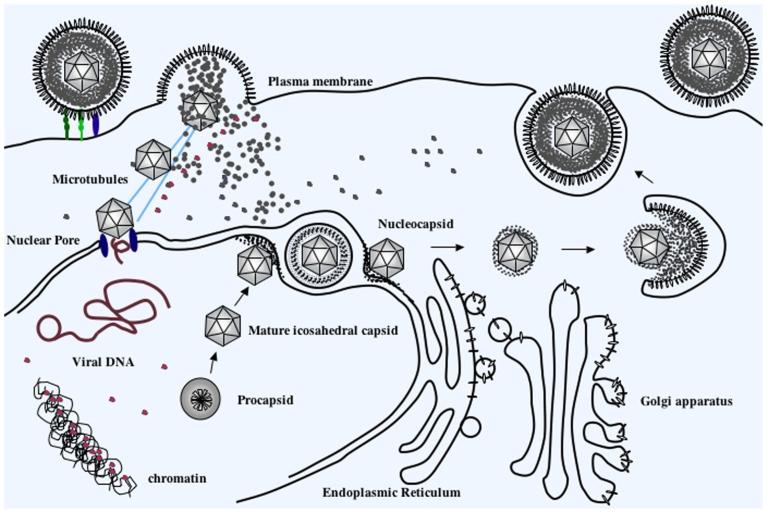
Schematic representation of localization of incoming VP22 during productive HSV-1 infection. Virion-derived VP22 (red spheres) enters in the cytoplasm of infected cells following binding of viral glycoproteins to cell-surface viral entry mediators and fusion of the viral and plasma membranes. Viral capsids follow microtubules (blue lines) to nuclear pores and inject viral genomic DNA into nuclei. Incoming, virion-derived VP22 enters nuclei and associates with chromatin. These events precede de novo VP22 synthesis, pro- and mature capsid formation, nucleocapsid egressing from the nucleus, virion re-envelopment in the cytoplasm, and vesicular migration to the cell surface (arrows).

As described in the Introduction, there exist many experimental data which are consistent with VP22 functioning in later stages during HSV-1 replication (Figure 6). The abundant accumulation of VP22 in infected nuclei in the late stages seems to suggest a role for VP22 in primary envelopment in the nucleus. The association of VP22 with viral glycoproteins and other tegument proteins fits with a role for VP22 in the re-envelopment phase of virion assembly. Our new data suggest a novel role for VP22 in the very first stage of infection. We show that virion-derived VP22 directly enters the nucleus and associates with chromatin.

## Data Availability

Data are contained within the article.
